# Biocompatible Composite Filaments Printable by Fused Deposition Modelling Technique: Selection of Tuning Parameters by Influence of Biogenic Hydroxyapatite and Graphene Nanoplatelets Ratios

**DOI:** 10.3390/biomimetics9030189

**Published:** 2024-03-20

**Authors:** Aura-Cătălina Mocanu, Andreea-Elena Constantinescu, Mădălina-Andreea Pandele, Ștefan Ioan Voicu, Robert-Cătălin Ciocoiu, Dan Batalu, Augustin Semenescu, Florin Miculescu, Lucian-Toma Ciocan

**Affiliations:** 1 Department of Metallic Materials Science, Physical Metallurgy, National University of Science and Technology POLITEHNICA Bucharest, 313 Splaiul Independentei, J Building, District 6, 060042 Bucharest, Romania; mcn_aura@hotmail.com (A.-C.M.); andreeaelena01c@gmail.com (A.-E.C.); ciocoiurobert@gmail.com (R.-C.C.); dan_batalu@yahoo.com (D.B.); 2 Department of Analytical Chemistry and Environmental Engineering, National University of Science and Technology POLITEHNICA Bucharest, 1-7 Gh. Polizu Str., 011061 Bucharest, Romania; pandele.m.a@gmail.com (M.-A.P.); svoicu@gmail.com (Ș.I.V.); 3 Advanced Polymer Materials Group, National University of Science and Technology POLITEHNICA Bucharest, 1-7 Gh. Polizu Str., 011061 Bucharest, Romania; 4 Department of Engineering and Management of Obtaining Metallic Materials, National University of Science and Technology POLITEHNICA Bucharest, 313 Splaiul Independentei, J Building, District 6, 060042 Bucharest, Romania; asemenescu2002@yahoo.com; 5 Academy of Romanian Scientists, 3 Ilfov Str., District 5, 050044 Bucharest, Romania; 6 Prosthetics Technology and Dental Materials Department, “Carol Davila” University of Medicine and Pharmacy, 37 Dionisie Lupu Street, District 1, 020022 Bucharest, Romania; tciocan@yahoo.com

**Keywords:** PLA/HA/GNP composite materials, printable filaments, improved mechanical properties, HA/GNP ratio influence

## Abstract

The proposed strategy for the extrusion of printable composite filaments follows the favourable association of biogenic hydroxyapatite (HA) and graphene nanoplatelets (GNP) as reinforcement materials for a poly(lactic acid) (PLA) matrix. HA particles were chosen in the <40 μm range, while GNP were selected in the micrometric range. During the melt–mixing incorporation into the PLA matrix, both reinforcement ratios were simultaneously modulated for the first time at different increments. Cylindrical composite pellets/test samples were obtained only for the mechanical and wettability behaviour evaluation. The Fourier-transformed infrared spectroscopy depicted two levels of overlapping structures due to the solid molecular bond between all materials. Scanning electron microscopy and surface wettability and mechanical evaluations vouched for the (1) uniform/homogenous dispersion/embedding of HA particles up to the highest HA/GNP ratio, (2) physical adhesion at the HA-PLA interface due to the HA particles’ porosity, (3) HA-GNP bonding, and (4) PLA-GNP synergy based on GNP complete exfoliation and dispersion into the matrix.

## 1. Introduction

On the verge of ceaseless discoveries and development in the realm of the modern manufacturing industry, additive manufacturing (AM), also known as 3D printing, has remained in the spotlight of many important technological sectors and enables the reshaping of crucial components while employing various basic and customized materials [1,2]. When addressing the biomedical field, the latter aspect was mainly challenged based on the ability of AM techniques (e.g., fused deposition modelling, FDM) to break through geometric and aesthetic constraints, proper accuracy, and processing costs, features that could beneficially reflect on the specific requirements for each application [3,4]. Currently, the irreversible effects of congenital defects, chronic ailments, the rapid aging of the population, or accidental traumas have technology spinning in a quest for new materials allowing for more efficient use during medical interventions, less environmental harm, and less depletion of some natural resources in the long run [5,6,7,8,9].

It is important to note the crucial task of choosing a suitable main biomaterial (e.g., metallic, polymeric, ceramic, or any combination) for the synthesis of high-performance feedstock materials with tuning parameters required for the 3D-printing process of bone-like structures [2,10,11]. In a sustainable manner, attention has shifted to biodegradable polymers, among which poly(lactic acid) (PLA) of natural origin (derived from maize, beet, or sugarcane) was nominated as an effective alternative with unique physico-chemical features (e.g., thermal stability, mechanical strength, and overall non-cytotoxicity) [7,12,13,14] and validated by the Food and Drug Administration (FDA) [15,16]. However, recent scientific advances have stipulated that the intrinsic brittleness and hydrophobic and inert surface features of PLA alone have to be mended properly for cells’ attachment and proliferation [6,15,17,18]. Several surface modification methods (processing with electron beams, plasma, or lasers [17]) or the blending/incorporation of assorted filler/additive materials (e.g., bioceramic, metallic, or carbon-based materials) [19] were tested in order to address these drawbacks.

At this stage, the next frontier in the field of biomaterials is the development of innovative composite biomaterials that can function with existing hardware equipment while also meeting the needs of specialized applications. Thus, the optimum filler materials, as well as their accuracy, dispersion degree, proper interfacial bonding, and ratios, are still to be determined [19,20,21].

In order to replicate the natural composite architecture of human bone, ceramic materials, particularly the calcium phosphate (CaP) division, were designated for this purpose [22,23,24,25]. The biogenic facet was also followed in this study based on the facile and reproducible synthesis of hydroxyapatite (HA) from natural resources (e.g., bovine or fish bones [26,27,28,29], marble, or seashells [23,30,31]) due to its physico-chemical and biological affinity for and similarity to natural bone [6,27,30,32]. Nonetheless, structures composed of HA alone revealed unsuitable mechanical resistance and a slow degradation rate with minimum induced porosity over prolonged periods from implantation [1,33,34].

For creating advanced composite filaments, recent biomedical studies have exploited the beneficial interplay between PLA and synthetic spherical HA particles, acquired mainly through chemical surface modification (the addition of binders and/or impact modifiers), but not always with the expected outcomes [18,34,35,36,37]. In addition to the limited incorporation ratios of ceramic material, ranging from 3 to a maximum of 40 wt.%, the reported composites lacked a proper particle distribution in the polymeric matrix, which led to the formation of agglomeration sites [6,38,39], as well as the ability to preserve the filaments’ surface uniformity and constant diameter size [18,38], both mandatory requirements for printable materials [40,41,42]. As it is well known that the higher the admixed HA amount, the higher the chances for the formation of new bone nucleation sites [36,38], attempts were made to improve it [37], but they led to the emergence of undesired porosity at the PLA–particle interface, as well as an implicit diminished mechanical resistance [18,33,37].

Other engineering strategies for the development of composite filaments target the graphene family of materials due to their promising mechanical and biocompatible features, which are transferable to graphene-reinforced mixtures [12,14,34,43]. Graphene nanosheets/nanoplatelets (GNP) can be securely anchored and bonded to matrix particles as nano-reinforcements, allowing for continuous stress transmission during the deformation process [3,19]. However, graphene exfoliation, filler–matrix adhesion, dispersion, and orientation remain current problems given the predisposition of GNP to layer stacking and agglomeration [12,34]. While some studies have shown that graphene-reinforced PLA poses poor mechanical properties, others have reported that by changing the raster orientation or using larger nanoplatelets, its capabilities may be improved [2]. Similarly, researchers have investigated the benefits of integrating essential oils or chemically prepared agents for enhancing the dispersion degree for both PLA/GNP [12,44] and PLA/GNP/HA mixtures [34]. These studies reported that even though the proportion of GNP was reduced to 1–4 wt.% for PLA/GNP and to 0.01–0.1 wt.% for the PLA/GNP/HA samples, the improvements in the mechanical behaviour were signalled only at the lowest amounts of GNP, in opposition to what was expected.

We propose here the incorporation of the highest ratios accounted for so far of bovine bone-derived HA (in the 0–50 wt.% range) and micrometric GNP (in the 0–5 wt.% range) materials into the PLA matrix (prepared without binders or surface modifiers). The strategy comes in order to overcome, one by one, the abovementioned deficiencies and to clarify the governing tuneable parameters for the development of printable composite filaments. First of all, we established the optimum dimensional range (<40 μm) of the HA particles in our previously reported study [45]. Following this perspective, a complex in vitro cytocompatibility investigation was conducted for a range of precursor materials in ref. [46], which retrieved the most adequate, non-cytotoxic type of GNP reinforcement (grade M, nanoplatelets of micrometric dimensions). To the best of our knowledge, only one published work has disclosed the addition of naturally derived fishbone HA into the PLA matrix; however, the procedure, processing parameters, and conducted investigations were inadequately defined [33].

Hence, we aimed here for the development of a functional, facile, and reproducible method for the fabrication of PLA/HA/GNP filaments with direct applicability for the manufacturing of 3D-printed bone-like products required in the fields of reconstructive orthopaedics and dentistry. The novel vision depicted in this study targets for the first time the clear definition of several endpoints corresponding to the tuning parameters by which the extruded PLA/HA/GNP filaments developed through this method are unique. The singularities of the HA particles derive from the isolation and extraction technology of HA from bovine bones [26,28,32]. As such, the HA particles act here as a double agent—as bone-like CaPs and reinforcement material: (i) the physical adhesion at the HA/PLA interface is favoured by the highly porous facets of the HA particles, which increases the surface tension without generating pores; (ii) the polyhedral shape of the biogenic HA particles with sharp and rugged edges contributes to the air bubble burst (an influence found nowhere in the literature): no entrapped bubbles originate during sample preparation, as compared to the synthetic spherical HA particles effect, and thus no internal porosity was generated. The chemical bonding at the HA/PLA interface is a consequence of the polar groups interconnectivity from both materials [47]. The HA-GNP bonding is assured based on the facile electrostatic interactions between the highly negatively charged graphene sheets and positive Ca^2+^ ions [20]. More to the point, the PLA-GNP synergy is based on the complete exfoliation of the GNP in the polymer matrix, which maximizes the dispersion degree and the mechanical takeover between the two materials [2,12].

Herein, the programmed investigations testify in favour of the optimal and tuneable parameters for the development of composite filaments with printable features. In a logical flow, subsequent research studies designated for the complex in vitro evaluation of the optimized 3D-printed composite products, based on the results provided in this study, were recently published [48] and sustain the vision entailed here.

## 2. Materials and Methods

### 2.1. Precursor Materials

The initial PLA (natural coloured granules, Φ = 2 ± 0.05 mm; Merck KGaA, Darmstadt, Germany) and xGNP^®^ (grade M: nanoplatelets with thickness ~7 nm and Φ = 25 μm; XG Sciences Inc., Lansing, MI, USA) materials were bought locally and used without any pre-treatment (chemical or physical). Materials were fully investigated previously in ref. [46]. The biogenic bone-like HA material was isolated through an established conversion procedure of bovine bones based on three successive thermal treatments, as previously reported [27,28]. The resulted HA powder was further subjected to ball mill grinding and granulometric sorting (standard sieves with meshes of 40 → 200 μm). Only the most adequate size sort (i.e., <40 μm) was chosen here, as we already demonstrated it to be a key parameter [45].

### 2.2. Preparation of PLA/HA/GNP Composite Filaments

**Figure 1:**
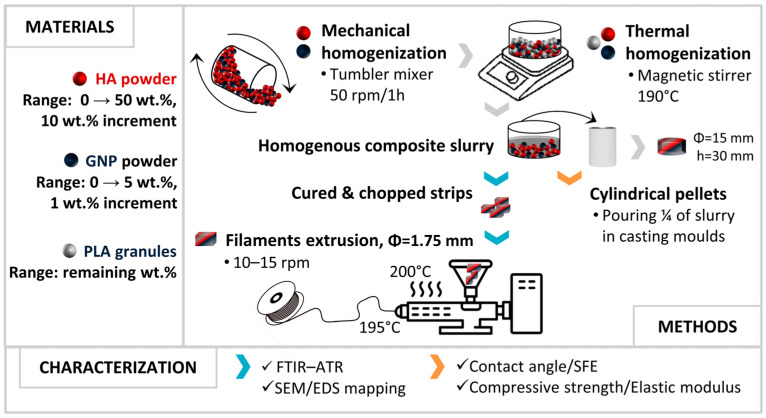
Graphical representation of the PLA/HA/GNP filaments fabrication procedure.

The preparation of composite filaments (Figure 1) required the incorporation of modulated ratios of HA (0–50 wt.% range; increment of 10 wt.%) and GNP (0–5 wt.% range; increment of 1 wt.%) into the PLA matrix. Hence, the sample codification was declared a function of the HA (e.g., 50% HA) and GNP (e.g., 5% GNP) ratios. The 100% PLA samples (0 wt.% HA, 0 wt.% GNP) were considered the control or reference materials. For each modulated HA/GNP ratio, the process involved the following steps: (1) the mechanical homogenization of the precursor materials for 1 h at 50 rpm in a tumbler mixer (Inversina, Bioengineering AG, Zürich, Switzerland); and (2) the thermal homogenization, at a constant temperature of 190 °C, of the resulting mixtures into the PLA matrix (remnant weight up to 100%), using a magnetic stirrer hob.

#### 2.2.1. Cylindrical Composite Pellets Destined for Wettability and Mechanical Investigations

From each sample type, ¼ of the obtained slurry was poured into casting moulds and allowed to cure and harden into cylindrical composite pellets (Φ = 15 mm, h = 30 mm). The plane-parallel surfaces of the composite pellets were acquired by grinding on abrasive paper (P400–2500). The pellets’ dimensions were set in compliance with the standardized requirements for an adequate evaluation of the wettability (samples with a diameter ≥ 10 mm according to ISO/TS 14778:2021 [49]) and the compressive strength (samples with a dimensionless ratio of diameter/length ≥ 0.4 according to ISO 604:2002 [50]) properties (both intrinsic material-dependent features [51,52,53]).

#### 2.2.2. Composite Filaments Extrusion

After curing and hardening, the remaining amount of each slurry was chopped and used as feedstock material for the extrusion of composite filaments. One extrusion cycle at 200 °C/195 °C (barrel/nozzle temperature) and 10–15 rpm (Pro Filament Extruder; Noztek, West Sussex, UK) for a uniform dispersion of both ceramic and GNP particles into the PLA matrix, followed by air cooling after emerging from the extruder nozzle (Φ = 1.2 mm), was performed. That is, the reported procedure here concerns only the already-determined optimum parameters for composite filament extrusion. A final estimation by weight revealed that from an initial 100 g of homogenized composite materials (regardless of the PLA/HA/GNP ratios), the total material loss varied between 3 and 5 wt.%, considering the final filament extrusion. The cross-section view of the composite filaments was exposed after cryogenic fracturing in liquid nitrogen.

### 2.3. Experimental Characterization Techniques

(a) FTIR-ATR Spectroscopy Measurements. The attenuated total reflectance (ATR) mode for Fourier-transform infrared (FTIR) spectroscopy was used to examine the chemical structure of all composite filaments. On a Bruker VERTEX 70 spectrometer (Bruker, Billerica, MA, USA), the FTIR-ATR spectra were obtained using 32 scans per sample, each obtained at a resolution of 4 cm^−1^ in the 4000–600 cm^−1^ area.

(b) Morpho-compositional Evaluation. The macro- and microstructure of the composite filaments, on both surface and cross-section view, were evaluated by scanning electron microscopy (ESEM Quattro™ microscope; Thermo Fischer Scientific, Hillsboro, OR, USA). The compositional features were determined with an auxiliary microanalysis EDS system (Thermo Scientific Pathfinder™). The micrographs’ acquisition was conducted on the extruded composite filaments in 5 randomly chosen areas (acceleration voltage = 15 kV, working distance = 10 mm) [54]. The distribution of the constituent elements was outlined through the EDS mapping technique.

(c) Wettability evaluation. The contact angle measurements were acquired on the cylindrical composite pellets using a Krüss Drop Shape Analyzer—DSA100 (A. Krüss Optronic GmbH, Hamburg, Germany). The experiments were performed with three different wetting agents (water, diiodomethane (DIM), and ethylene glycol (EG)) at constant room temperature (20 ± 1 °C) and humidity (45 ± 5%). The images were captured 1 s after the deposition of the wetting agent droplet. The results (average of 5 determinations/sample) were afterwards processed with the ImageJ 1.50 software (National Institutes of Health, Bethesda, MD, USA). The surface free energy (SFE) was computed based on the contact angle measurements through the OWRK (Owens, Wendt, Rabel, and Kaelble) method [55,56].

(d) Mechanical behaviour assessment. The compressive strength and elastic modulus of all cylindrical composite pellets were determined using a universal test machine (type LFV300; Walter + Bai AG, Loehningen, Schaffhausen, Switzerland) at a test speed of 1 mm/min and an acquisition rate of 0.01 s. The results represent the average of three sets of measurements.

## 3. Results and Discussion

### 3.1. FT–IR Evaluation

**Figure 2:**
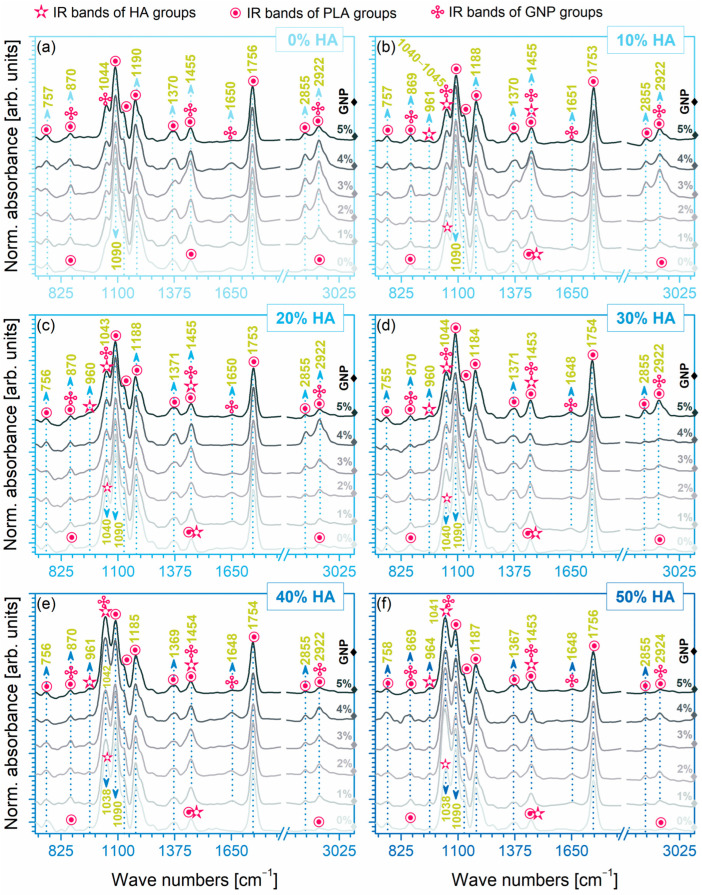
FTIR-ATR spectra of the extruded composite filaments: (**a**–**f**) PLA/HA (0–50 wt.%)/GNP (0–5 wt.%).

The FTIR-ATR fingerprints of the extruded composite filaments are comparatively displayed in Figure 2. The infrared spectral profiles revealed a favourable combination of overlapping bands in accordance with the HA/GNP incorporated amounts. As such, the FTIR identification recorded specific IR bands for GNP—only in the case of reinforced samples—along with specific bands attesting the presence of HA at ratios ≥10 wt.%, and as expected, bands with the highest intensity for the PLA matrix for all samples.

The molecular homogeneity was demonstrated for all sample sets by the identification of both specific and particular IR bands. As such, the reference sample (100% PLA) in Figure 2a reveals only the main characteristic bands of the PLA groups, namely: symmetric stretching of C=O (~757, ~1756 cm^−1^), symmetric (1090 cm^−1^) and asymmetric (1190 cm^−1^) stretching of C–O–C, symmetric stretching of CH_3_ (1453 cm^−1^), and symmetric (2855 cm^−1^) and asymmetric (~2922 cm^−1^) stretching of –CH groups [17,18,21,33]. Given that both raw PLA granules and PLA extruded filament outlined the deformation (bending modes) of the C–H and C–COO groups in the 1367–1370 and ~870 cm^−1^ regions, respectively [46], the hypothesis that the polymeric chain could suffer some modifications when subjected to high temperatures [57] during extrusion is excluded.

Moreover, for the PLA/GNP composite mixtures exposed in Figure 2a, the specific IR bands of the polymeric matrix were preserved, and particular vibrations of intensities dependent on the GNP ratio appeared and were ascribed to the symmetric stretching (double-split shoulder at 1044 cm^−1^) of C–O and the aromatic stretching (1645 cm^−1^) of C=C groups. The other vibrations, corresponding to the aromatic deformation of the C–H groups (870 cm^−1^), and of the bending mode of the hydroxyl (–OH) functionalities (slightly shifted to 1455 cm^−1^), and the symmetric stretching of CH_2_ groups (~2922 cm^−1^) in pristine GNP [58,59,60], were juxtaposed with some associated PLA functional bands. This behaviour reflects directly on the grafting mechanism of the PLA chains onto the surface of GNP. Some studies have reported in this regard that due to the shifting of the bonds, the peaks with the higher intensity at 1753–1756 cm^−1^ (GNP ≥ 1%) could also be a consequence of the grafting reaction between the two materials [58].

Similarly, in the case of the PLA/HA composites (Figure 2b–f with indicated 0% GNP), the presence of the HA adsorption bands lay in the ranges for the corresponding (PO_4_)^3−^ group vibrations: the symmetric (~961 cm^−1^) and asymmetric (~1040–1044 cm^−1^) stretching. In contrast to the crystalline commercial HA, the naturally derived one elicited specific IR spectra assigned to the asymmetric stretching modes of carbonate groups (centred at 1455 cm^−1^), as predicted and reported in prior investigations [27,28,31], which were superimposed with the PLA afferent vibration band.

Consequently, when both HA and GNP were incorporated at modulated ratios into the polymeric matrix (Figure 2b–f with indicated 1–5% GNP), the frequency assignment depicted a second level of overlapping due to the solid bond formation and interference at the molecular level between all types of materials. While some bands remained unchanged across all spectra, due to the increased uptake of the HA component at 40 and 50 wt.%, some of the PLA band (e.g., 1090 cm^−1^ and 1756 cm^−1^) intensities decreased significantly in favour of the (PO_4_)^3−^ groups in HA emerging at bands slightly shifted to 1038 cm^−1^, regardless of the GNP ratio.

### 3.2. SEM/EDS Evaluation

**Figure 3:**
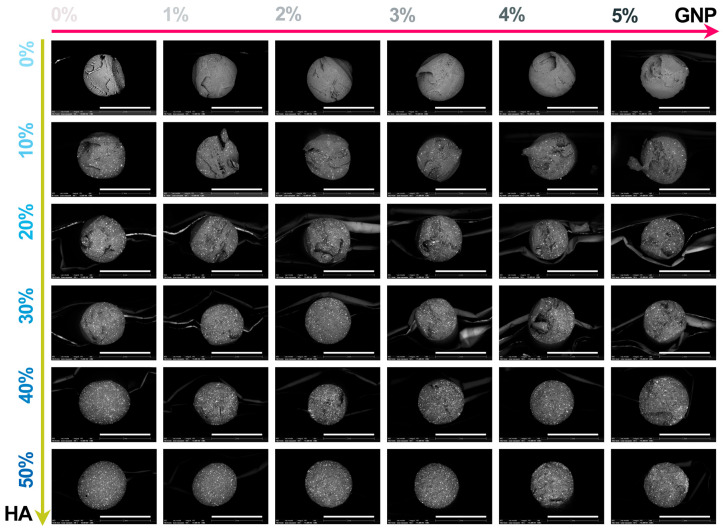
Representative macroscopic images acquired in cross-section view of the extruded composite filaments: PLA/HA (0–50 wt.%)/GNP (0–5 wt.%). Scale bar: 2 mm.

**Figure 4:**
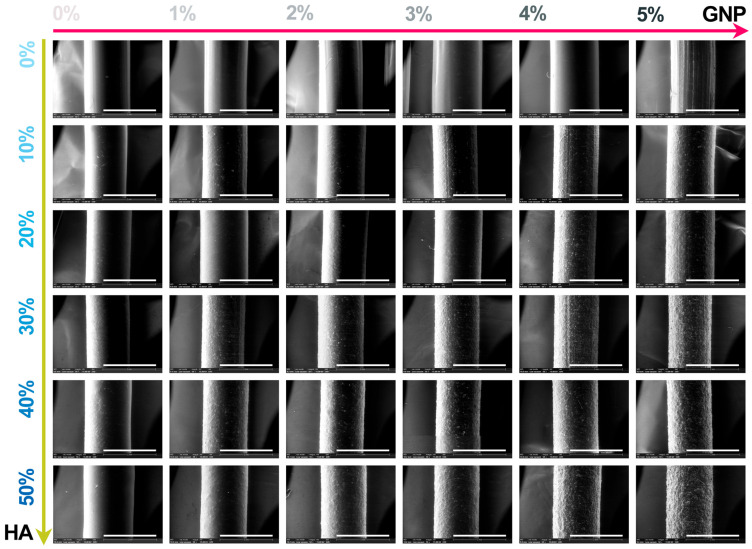
Representative macroscopic images acquired on the outer surface of the extruded composite filaments: PLA/HA (0–50 wt.%)/GNP (0–5 wt.%). Scale bar: 2 mm.

The macroscopic results for all extruded composite filaments prepared with modulated ratios of HA and GNP as reinforcement materials are presented in Figure 3 and Figure 4, respectively. The assessment, comprising the cross-section view and outer surface (top-view) of the samples, was carried out in order to disclose the influence of the GNP addition upon the fine and uniform dispersion degree of the ceramic component into the polymeric matrix and their conjoined impact upon the overall integrity and full-length uniformity of the filaments.

The macrographs acquired in cross-section view revealed the homogeneity of the composite filaments after extrusion through the even scattering of the ceramic particles into the polymeric matrix, independent of the HA and GNP ratios. Moreover, the non-preferential dispersion of the ceramic filler also reached the outer surface of the filaments. Here, the reference sample presented a smooth and neat surface that changed once the GNP ratio seized, gradually, the highest value, leading to the formation of straight indentations on the outer shell. The phenomenon appeared more pronounced with the successive addition of HA particles in higher ratios, due to the supplementary emergence of mostly round and micrometric irregularities on the filaments’ surface. At the intersection of high loading concentrations of the reinforcement materials (above 30% HA and 3% GNP), larger and rougher protuberances patterned the surface.

However, this behaviour could be significantly related to the increment of the van der Waals forces between the graphene platelets once their ratio increased, leading to the enhanced overlapping and thickening of the sheets [34]. This can also be tracked at higher magnification scales.

**Figure 5:**
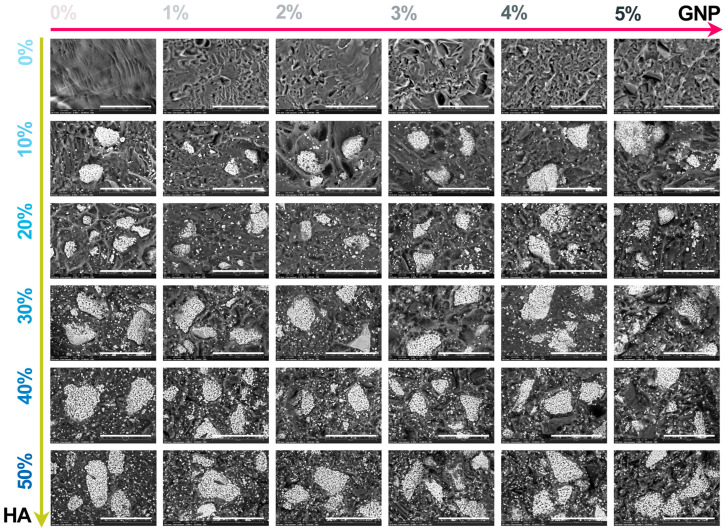
Morphological evaluation in cross-section view of the extruded composite filaments: PLA/HA (0–50 wt.%)/GNP (0–5 wt.%). Scale bar: 50 μm.

**Figure 6:**
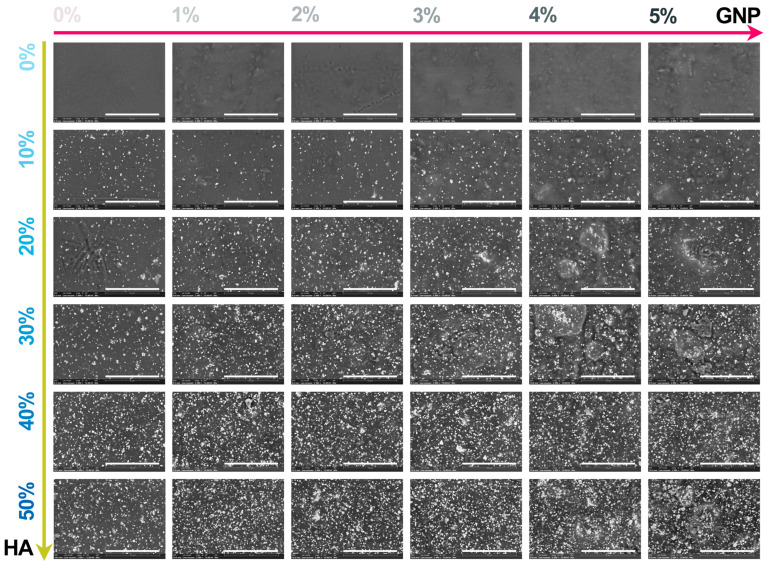
Morphological evaluation on the outer surface of the extruded composite filaments: PLA/HA (0–50 wt.%)/GNP (0–5 wt.%). Scale bar: 50 μm.

Hence, the micrographs of the extruded filaments analysed in both cross-section (Figure 5) and top view (Figure 6) confirmed the incipient findings and exposed supplemental features afferent to the HA and GNP ratios. Here, the fracturing evolved from brittle-like behaviour accompanied by folds of wavy lines for the reference sample and PLA/HA (up to 30%) samples to a more ductile predisposition for samples with the gradual incorporation of GNP (up to 3%), showcasing the existence of fibril structures (Figure 4). However, once the loading ratios exceeded these values, the composite filaments presented a mixed morphology that evolved to microfracturing at the highest HA/GNP ratio.

For all extruded composite filaments, a fairly uniform dispersion and homogenous embedding of the micrometric ceramic particles into the polymeric matrix were depicted, while progressive surface coverage was assured by the larger polyhedral particles once the HA ratio increased. During the extrusion process, most of the particles’ edges were reformed from sharp to round, but the particles’ specific morphology, composed of micrometric pores [26], was preserved and clearly outlined for the larger particles. Thus, their inherent microporosity and improved specific surface area stand as favourable factors for a strong adhesion at the polymeric–ceramic interface, otherwise possible only by chemical routes [18].

In contrast, wide pores and voids, which originated with the addition of GNP into the polymeric network, were sequentially enclosed by the embedment of the HA particles. Even so, this sympathetic mechanism functioned until the highest HA/GNP loadings were involved (that is, 30–40% HA and 4–5% GNP). At this stage, remnant voids, pores, and nuclei of agglomerated and compactly encapsulated particles in the form of folds were delineated into the PLA matrix, possibly due to the poor dispersion at high GNP ratios [34]. This effect was even more distinctively presented by the correspondence of folds→ micrometric bumps, scattered in an irregular manner on the outer surface of the filaments (Figure 6). Due to the overlapping of numerous bumps, several protuberances of varied dimensions were depicted in each micrograph and could serve as stress concentrators, leading to low mechanical uptake.

In terms of using the composite mixtures as feedstock materials for the 3D-printing process, the later observed features may come across as incompatible with the specific requirements for constant filament diameter and surface–volume homogeneity (both essential for the prevention of material clogging in the printer nozzle) [6,40,41].

Therefore, with the precise modulation of the precursor material ratios and the adjustment of the nozzle diameter and extrusion temperature, the resulting composite filling could properly respond to the desired and mandatory quality attributes of the final products [38].

**Figure 7:**
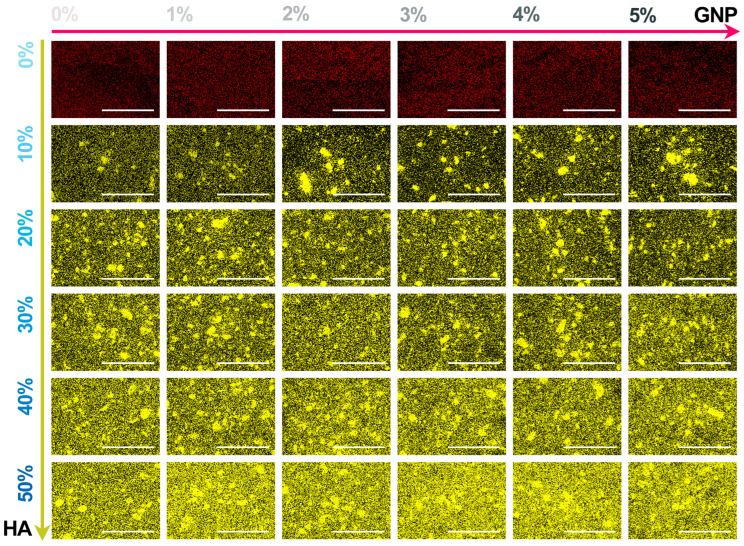
Compositional evaluation of the extruded composite filaments: PLA/HA (0–50 wt.%)/GNP (0–5 wt.%). Chemical elements: C (red colour); Ca (yellow colour). Scale bar: 200 μm.

The comparative representation of the elemental mapping, performed in cross-section view for all extruded composite filaments, is given in Figure 7. The balance of the main chemical elements was selected based on their relevance to the sample type: with or without HA incorporated into the ceramic matrix [27,45,46]. Thus, for the reference and PLA/GNP samples, the C content (red colour) presented a predominantly uniform dispersion across the micrographs, regardless of the GNP ratio.

Concomitant with the addition of HA particles, the characteristic Ca content (yellow colour) presented a cvasiuniform distribution inside the filaments, outlining the ceramic particles of all sizes (in the <40 μm range) and the afferent proportional degree of coverage, which supports the SEM findings (Figure 5). Moreover, dependent on the gradually increased HA ratio, the Ca content intensity spiked in constant upward shifts, followed row by row in Figure 7. Correspondingly, for all samples, the dark background was attributed to the O content. Hence, a uniform dispersion of HA particles was also endorsed based on the difference in atomic number as compared to the components of the polymeric matrix.

### 3.3. Contact Angle and Surface Energy Investigations

**Figure 8:**
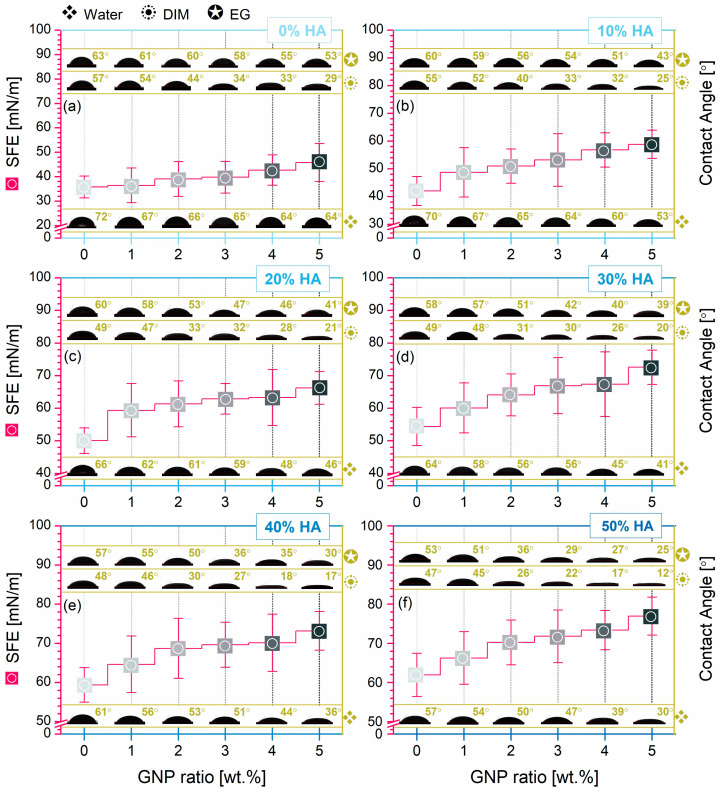
Surface wettability by contact angle measurements (wetting agents: water (W), diiodomethane (DIM), ethylene glycol (EG)) and surface free energy (SFE) computed by the OWRK method for all composite pellets: (**a**–**f**) PLA/HA (0–50 wt.%)/GNP (0–5 wt.%).

With far-reaching significance to the final envisioned biomedical applications of the developed composite materials and directly correlated to adequate cellular behaviour and response after implantation, their wettability degree and surface free energy (SFE) variations are shown in Figure 8. The surface hydrophilicity was evidenced by contact angle measurements (CA), with three wetting agents (water (W), diiodomethane (DIM), and ethylene glycol (EG)) used as parameters for the dispersive and polar interactions at the solid–liquid interface required for further computing the SFE through the OWRK method [56,61]. The nature of each wetting agent is known and reported [56]; here, water and EG were employed as polar components, whereas DIM was a nonpolar or dispersive one.

Considering the PLA and PLA/HA samples as platforms for comparison, the results steered a clear favourable influence of each reinforcement material, in turn, upon the wetting behaviour of the composite pellets. It was noticed that for both instances, the CA values dropped progressively once the HA and GNP were incorporated at higher ratios (in the investigated ranges), independent of the wetting agent. For the PLA—PLA/HA set, the W contact values ranged between 72° and 57°, while the DIM and EG ones ranged between 57° and 47° and 63° and 53°, outlining an elevated wettability disposition (CA < 90°) of the composite materials with modulated HA addition compared to the PLA alone.

The surface feature augmentation was further validated by the drastic exponential decrease in the CA values with the increment of the admixed GNP ratio to the PLA/HA materials. The incipient stage of the GNP mechanism was observed for the PLA/GNP samples, where the DIM value was halved and the others decreased by ~0.88 → 0.84 (for W and EG, respectively), corresponding to the maximum GNP amount. This is in complete contrast to portrayal in the literature of the GNP materials as water barrier effect consolidators due to their inorganic and impermeable state [12]. When both reinforcement materials reached the highest HA/GNP ratio, the CA values conveyed the lowest records compared to the PLA reference (downturn by ~0.42, ~0.21, and ~40 for W, DIM, and EG, respectively). The corresponding SFE values corroborated the CA findings, retrieving an opposite trend line of the results for both HA and GNP addition in turn and concomitantly. Thus, both indicators argued towards a deep-seated wettability of the composite pellets, as was expected given the surface morphology (a key factor for the wettability evolution) composed of micrometric bumps, increased concentration, and regular dispersion of the embedded porous ceramic particles in any section of the samples (see Figure 5 and Figure 6), leading to progressively rougher surfaces [15]. Furthermore, particular attention was given to the balance between these governing factors in additive manufacturing for properly printing continuous lines without the full-merging phenomenon and for a given resistivity [13,62]. Moreover, the surface wettability of the composite materials will further influence the overall wetting behaviour, integration, and degradation degree of the 3D-printed products after implantation [52,63].

Overall, the obtained results translate into enhanced protein adsorption and the positive cellular responses (adhesion, proliferation, and survival) required for new bone formation at the composite materials–host tissue interface, the paramount goal in the bone reconstruction applications field [7,13,39,64,65].

### 3.4. Mechanical Features Evaluation

**Figure 9:**
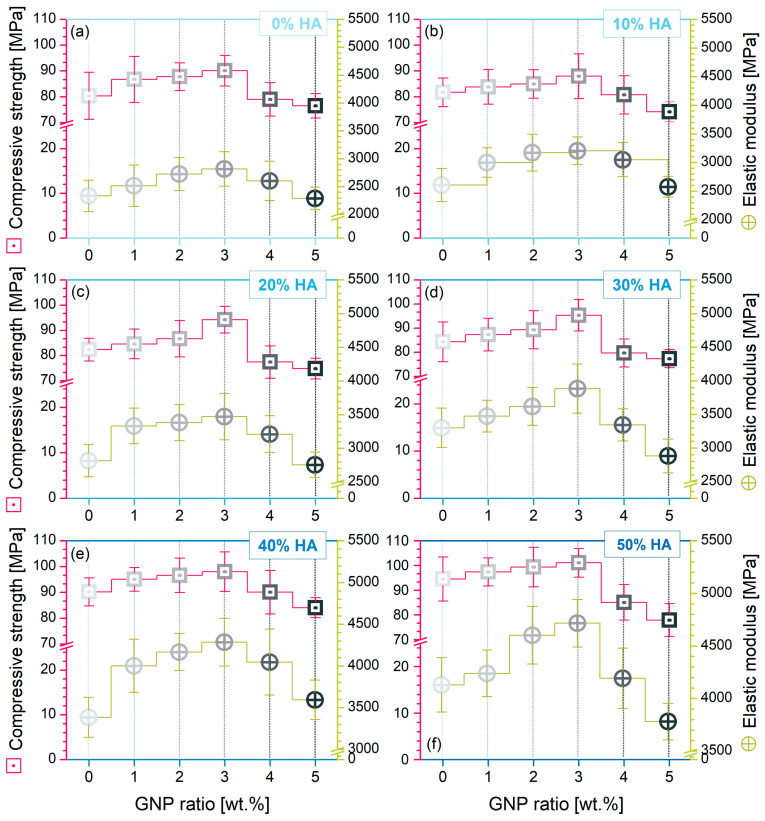
Compressive strength and elastic modulus variation for the composite pellets: (**a**–**f**) PLA/HA (0–50 wt.%)/GNP (0–5 wt.%).

The mechanical behaviour of all composite pellets was evaluated by uniaxial compression testing. The afferent mean values and standard deviations for the compressive strength and elastic modulus are graphically presented in Figure 9.

At first glance, one can easily note that the evolution line for the mechanical features was preserved across all samples and was directly linked to the modulated HA/GNP ratios. Considering the same comparison platforms as above mentioned (PLA and PLA/HA samples), the variation in compressive strength and elastic modulus outlined a linear upward trend line with the increase in the incorporated GNP ratio in the 0–3 wt.%, while for the next 4–5 wt.% increments, the records disclosed an abrupt downshift trend, regardless of the HA ratio. This backsliding tendency could be attributed to a series of governing factors related to the morphology, dispersion degree, and intrinsic characteristics of GNP. Hence, previously discovered pores, voids, and microcracks (Figure 4) directly impacted the mechanical strength, as it is well known that the higher the remnant porosity or void volume, the lower the final mechanical performance [43,66]. Another potential factor with dramatic consequences in this regard refers to the lack of dispersion degree of GNP at high loadings, arising in the form of folds or protuberance networks accompanying the HA particles and acting against adequate stress transmission throughout the samples [12,34]. Another intertwined aspect in this direction could be the stacking effect of the GNP sheets due to the inferred strong van der Waals interactions [34]: a certain maximal amount can be loaded for attaining optimal results.

Compared to PLA alone, the addition of HA in the 10–50 wt.% range led to the improvement of the compressive strength and elastic modulus by ~1.02 → 1.18 and ~1.12 → 1.77, respectively. However, when GNP (0–3 wt.%) was loaded into the polymeric matrix, the compressive strength and elastic modulus were further enhanced by ~1.08 → 1.12 and by ~1.08 → 1.21, respectively. This suggests a better play for the mechanical reinforcement mechanism on the GNP account. These results are in tune with other reports concerning the effect of graphene fillers on mechanical behaviour, due to their greater tensile strength and elastic modulus, which block the movement of the polymeric chains under strain or load-bearing actions [2,13].

Outstanding results were acquired for samples with conjoined loaded HA/GNP ratios, as their in turn interplay and connection with the polymeric matrix could explain the exhibited higher variation in compressive strength and elastic modulus (by ~1.18 → 1.26 and by ~1.77 → 2.02, respectively), compared to the PLA values. Here, the results state the following: (1) the physical adhesion at the HA-PLA interface was favoured by the highly porous facets of the ceramic particles, which increase the surface tension without generating supplementary pores [37], followed by the chemical bonding of the polar groups from both materials [47]; (2) the HA-GNP bonding occurred through the electrostatic positive–negative charges and interfacial interactions of graphene sheets with Ca^2+^ ions [20,67]; and (3) the PLA-GNP synergy was based on the GNP complete exfoliation in the polymer matrix predicted at lower ratios, which could maximize the uniform dispersion degree and impact stress takeover between the two materials [2,12,34].

Considering the incorporation of naturally derived HA and GNP materials at modulated ratios, all three prerequisites were met for the further development of products with a greater resemblance to the natural bone behaviour—optimal mechanical results were favourably delineated according to the requirements for bone reconstruction applications (cortical and trabecular bone-like performance) [6,68,69,70]. Moreover, when needed, the surface, composition, and mechanical features can be adapted and patiently customized by tuning the reinforcement material ratio.

## 4. Conclusions

The feasibility of extruding composite PLA/HA/GNP filaments through the envisioned strategy was confirmed. The influence of the conjoined incorporation and modulation of the highest ratios of reinforcement materials (HA, GNP) into the PLA matrix on the molecular architecture, morphology, wettability, surface free energy, and mechanical behaviour were accounted for here for the first time. The results of the correlation pinpointed the optimal HA/GNP ratio required for the synthesis of printable composite filaments for biomedical applications.

When both HA and GNP were incorporated at modulated ratios into the polymeric matrix, the FTIR frequency assignment depicted two levels of overlapping structures vouching for solid bond formation at the molecular level between all types of materials, free of any traces, impurities, or other molecules or compounds.

Due to the technological conversion of bovine bones into HA, the singularities of the derived ceramic particles acted as a double agent by (1) supporting the physical adhesion at the HA/PLA interface due to the induced highly porous facets and (2) contributing to the bursting of air bubbles (usually entrapped into the polymer during preparation) and to no generated internal porosity due to their polyhedral shape with sharp and rugged edges. Further, due to the synergy between the HA-GNP and PLA-GNP components, the overall mechanical features signalled an enhancement limit corresponding to the GNP modulation up to a maximum 3 wt.%, regardless of the HA ratio, leading to bone-compatible compressive strength and elastic modulus values. Furthermore, the hydrophilic nature outlined for all composite samples revealed the HA/GNP potential to induce positive outcomes in terms of PLA’s improved wettability and future cell responses, independent of the modulated ratios.

Overall, the proposed tuneable design for the synthesis and extrusion of suitable composite reinforced filaments enables their use as feedstock materials for AM technologies, with close regard to the bone application requirements.

## Data Availability

Data are contained within the article.
